# Mechanisms and control measures of low temperature storage-induced chilling injury to solanaceous vegetables and fruits

**DOI:** 10.3389/fpls.2024.1488666

**Published:** 2024-11-11

**Authors:** Qi Yuan, Yaqin Jiang, Qihong Yang, Weiliu Li, Guiyun Gan, Liangyu Cai, Wenjia Li, Chunchun Qin, Chuying Yu, Yikui Wang

**Affiliations:** ^1^ Vegetable Research Institute, Guangxi Academy of Agricultural Sciences, Nanning, China; ^2^ College of Agriculture, Guangxi University, Nanning, China

**Keywords:** solanaceae, low temperature storage, chilling injury, resistant mechanisms, preventive measures

## Abstract

Low temperature storage is widely used for storage and transportation of fruits and vegetables after harvest. As a cold-sensitive fruit vegetable, post-harvest solanaceous vegetables and fruits are susceptible to chilling injury during low temperature storage, which reduces its sensory quality and edible quality and shortens its storage period, thus leading to huge economic losses. Therefore, it is an essential to clarify the occurrence mechanism of chilling injury caused by low temperature storage in solanaceous vegetables and fruits, and to propose corresponding prevention and control measures for chilling injury. In recent years, a series of progress has been made in the research on chilling injury prevention and control and low temperature stress tolerance of solanaceous vegetables and fruits. This paper describes the chilling injury symptoms of postharvest solanaceous vegetables and fruits, clarifies the physiological and biochemical mechanisms in the chilling injury process, the molecular mechanisms, and prevention and control measures, and summarizes the latest research advancements on chilling injury and chilling tolerance regulation of solanaceous vegetables and fruits, which can provide valuable references for low temperature storage and chilling injury prevention and control measures of solanaceous vegetables and fruits.

## 1 Introduction

Solanaceous vegetables are horticultural species with important economic significance, including tomatoes (*Solanum lycopersicum* L.), peppers (*Capsicum annuum*), eggplants (*Solanum melongena* L.), and potatoes (*Solanum tuberosum* L.). Solanaceous fruits and vegetables are rich in nutrients and have unique flavors, and they are widely consumed around the world. However, their short shelf life and large post-harvest losses affect their economic value. Low-temperature refrigeration is widely used in post-harvest storage and preservation of horticultural products, and it can decrease the metabolic rate of plant cells, delay senescence, reduce decay, and maintain the quality of horticultural products. However, solanaceous fruits and vegetables belong to tropical and subtropical fruits and vegetables. During storage and transportation, long-term exposure to low temperatures could cause chilling injury (CI) to fruits and vegetables, especially to solanaceous vegetables that are very sensitive to temperatures below 12°C, which led to a series of physiological disorders, such as immaturity, softening, decay, and flavor deterioration (Liu Y. D. et al., 2023). According to statistics, cold-sensitive fruits and vegetables account for 50% of total fruit and vegetable products, and their annual post-harvest economic losses reach hundreds of billions of yuan, of which the losses caused by CI account for more than 1/3 (Zhang and Jie, 2016). The CI problem severely restricts the development of fruit and vegetable industry in China. The exploration of mechanisms of CI in solanaceous fruits and vegetables and effective measures to reduce CI has become the research hotspot of fruits and vegetables.

In recent years, with the development and application of modern molecular biology technology, in-depth research has been conducted on the mechanisms underlying CI caused by low-temperature storage of fruits and vegetables and the corresponding regulations from multiple perspectives such as molecular biology, genomics, proteomics, cell biology, physiology, and biochemistry (Zhang W. L. et al., 2021). Therefore, this paper aims to outline the latest progress in the biological mechanisms and prevention and control methods of CI in solanaceous fruits and vegetables, and present future directions for the further research on CI of solanaceae horticultural products.

## 2 Influencing factors and symptoms of CI

### 2.1 Influencing factors of CI

**Figure 1:**
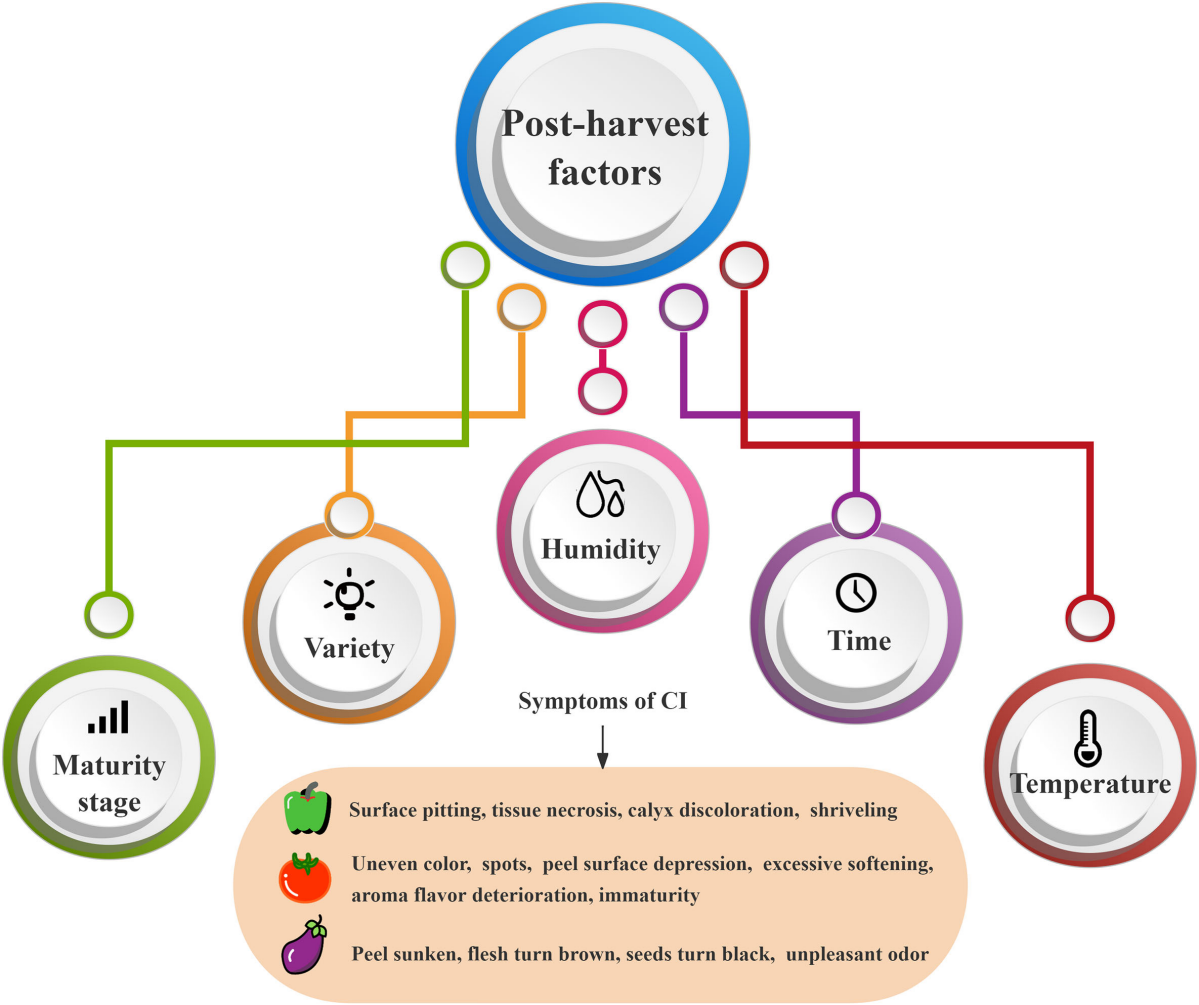
Influencing factors and symptoms of postharvest CI in solanaceous vegetables and fruits.

Chilling injury refers to the physiological damage to chilling-sensitive fruits and vegetables, when they are stored at inappropriately low temperatures above freezing point. The critical temperature of CI varies with different horticultural products. For example, the chilling-sensitive temperature of subtropical fruits and vegetables is 5°C~8°C, while that of tropical fruits and vegetables is below 12°C (Yao et al., 2018). CI can occur at all stages of plant growth and development, pre-harvest factors genotype, environmental variables, and agronomic practices, all interact to influence CI severity (Yahia et al., 2019), thus should be considered for their importance in the development of postharvest quality. However, the most significant effects on post-harvest were internal factors (variety and maturity stage) and external factors (temperature, humidity and time), as shown in **Figure 1**. Purple long eggplant and black round eggplant showed CI only after 3 days of storage at 2°C, and the CI degree of purple long eggplant is more serious at the same temperature (Guo et al., 2016). Many studies have shown that the maturity of solanaceous fruits and vegetables at harvest also has an important effect on the occurrence of CI. For example, tomatoes are also very sensitive to low temperatures, and in green maturity period, their storage below 10°C will cause CI, but the storage above 13°C will not (Biswas et al., 2016), while in red maturity period, CI occurs only at the storage below 5°C (Zhao et al., 2009). Similarly, the sweet pepper storage above 7.5°C in green maturity period can prevent the risk of CI (Yeboah et al., 2023). Among the external factors, temperature and storage time are the most important regulatory conditions. Gao et al. (2015) have reported that the first symptoms of CI to eggplant fruit appeared within 5 days of storage at 1°C, in the form of irregular pitting of the fruit skin, more severe CI was observed on day 15. The longer the pepper is stored at 4°C, the chilling injury index reaches 50%, and the greater the loss of chlorophyll and vitamin C content (Wang Y. X. et al., 2019). Interestingly, Zhao et al. (2003) studied the postharvest storage of chili fruits at four different temperatures, 10°C, 7°C, 4°C, and 1°C, and found that with the prolongation of the storage time, the symptoms of cold damage were more pronounced, especially in the chili fruits at 4°C, where the cold damage occurred earlier and to a more serious extent. Additionally, relative humidity is also an important factor affecting the occurrence of CI, which is reflected by the changes in moisture during the storage of fruits and vegetables. Afolabi et al. (2023a) researched sweet peppers were stored at 5°C with a RH of 98 ± 2% and 70 ± 6% for high and low RH, respectively, and results found that high RH storage reduced fruit water loss by 4%~4.5% compared to low RH storage, resulting in fewer CI symptoms regardless of fruit maturity stage. Similarly, Zhang M. et al. (2021) compared the cold damage of chili peppers in different humidity environments at the same temperature (4°C) and found that the cold damage index of the high humidity group (96%-99%) was 37.04%, which was 54.54% lower than that of the low humidity group (70%-75%). In addition, the storage with humidity controlled by dry fog inhibits the production of reactive oxygen species (ROS) in eggplants and increases the activity of antioxidant enzymes, thus significantly reducing the CI index of eggplants (Cao et al., 2021).

### 2.2 Symptoms of CI

When fruits and vegetables are stored at room temperature after harvest, they will produce a large amount of ethylene, leading to rapid aging, rot, and quality deterioration, thus resulting in economic losses. Low-temperature storage can minimize the loss of color, flavor, and texture qualities, hence delaying aging and preventing decay. Therefore, the widespread use of refrigeration can ensure the quality of fruits and vegetables throughout the entire supply chain. However, improper low temperature storage can cause CI symptoms such as peel softening, surface depression, peel and flesh browning, aroma deterioration, seed blackening, and abnormal ripening (**Figure 1**), which seriously affects the sensory quality and edible quality of fruits and vegetables.

Solanaceae is a tropical and subtropical cold-sensitive vegetable, and low temperature storage will cause CI to it. For example, tomatoes in green maturity stage stored at 12°C show CI symptoms such as uneven color, spots, peel surface depression, excessive softening, aroma and flavor deterioration, and immaturity (Aghdam et al., 2019), and they are also susceptible to the infection by *Alternaria* fungus, resulting in more serious CI such as rot. When eggplant was stored at 7°C~10°C, the peel was sunken; the flesh became brown; and the seeds became black, with an unpleasant odor (Huang et al., 2019). As for sweet peppers, the storage below 7°C resulted in soft fruit surface, sunken spots, and the browning of seeds and calyx (Fu et al., 2022). The CI causes the metabolic imbalance in tomatoes, eggplants, and peppers, further leading to physiological diseases, a decrease in sensory quality and commercial value, or even serious deterioration in the quality of solanaceous fruits, thereby completely losing the original fruit flavor.

## 3 Physiological and biochemical mechanisms of CI

**Figure 2:**
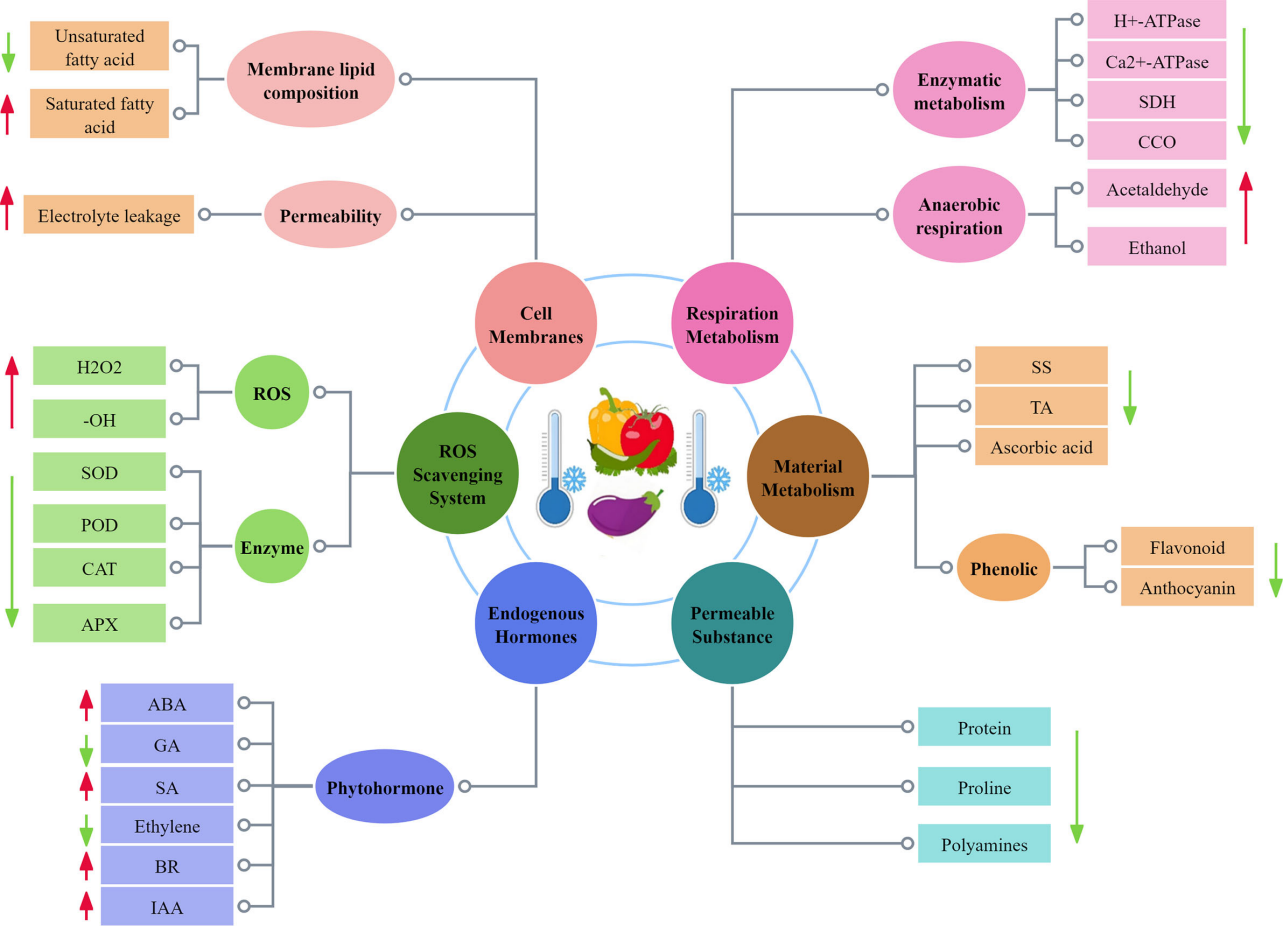
Physiological and biochemical changes of CI in solanaceous vegetables and fruits.

Under low-temperature stress, a series of changes occur in the physiology of fruits and vegetables, as shown in **Figure 2**. In recent years, it has been reported that the physiological and biochemical mechanisms of CI in fruits and vegetables include cell membrane damage and free radical damage. Cell membrane damage is the most primary factor causing CI in fruits and vegetables, followed by physiological and metabolic disorders. In contrast, biological free radicals have a strong destructive effect on biological macromolecules such as chloroplasts, proteins, and nucleic acids of cells. After fruit and vegetable tissues are subjected to CI, the ability to scavenge free radicals from fruits and vegetables is weakened, and free radicals can accumulate excessively, leading to the destruction of biological macromolecules (Zhang W. L. et al., 2021).

### 3.1 Effects of CI on cell membranes

Membranes are the most important structures in cells, playing an important role in the sensing and barrier functions of cells, and they also enable various physiological and biochemical reactions to proceed in an efficient and orderly manner. When fruits and vegetables encounter extreme conditions such as cold stress, the membrane system change its physical phase from a flowing liquid to a solid gel, and this transition decrease the fluidity of the cell membrane and even changes its structure, thus preventing cell membrane from performing normal physiological functions (Wang, 1994).

Low-temperature stress directly leads to a large amount of electrolyte extravasation in plant cells, so the rate of electrolyte extravasation is usually used to characterize the disruption of cell membrane permeability in fruits and vegetables. Electrolyte leakage is an effective parameter to evaluate the membrane permeability of fruits and vegetables during the cold storage period (Meng et al., 2012). Barzegar et al. (2021) found that eggplants stored at 7°C (7, 14, and 21 d) had greater electrolyte leakage the longer it was stored. Similarly, CI occurred in bell peppers during low-temperature storage (5°C and 12°C), where the selective permeability of the cell membrane was reduced, and the leakage of ions from the membrane to the outside of the membrane increased significantly with the extension of the storage time (Gabriela et al., 2021). These findings suggest that low temperatures cause severe physical damage to the cell membrane structure of fruits and vegetables, leading to increased cell membrane permeability, resulting in increased cellular extravasation of substances and increased CI.

When fruits and vegetables are subjected to CI during cold storage, the membrane lipid composition will undergo significant changes, which is mainly indicated by changes in membrane phospholipid composition and fatty acid content. CI of green pepper has been reported to reduce the total level of membrane lipids, which is mainly manifested in a decrease in the contents of monogalatosyl diglyceride (MGDG), phosphatidyl cholines (PC), and phosphatidylethanolamine (PE), but the contents of phosphatidic acid (PA) and lysolecithin increase (Kong et al., 2018). Zhang X. Y. et al. (2021) reported that the contents of MGDG, digalactosyl diglyceride (DGDG), PC, PE, phosphatidylglycerol (PG), and phosphoinositide (PI) in green pepper fruits decreased during cold storage, while the contents of PA and lysophospholipids increased, that the contents of palmitic acid and stearic acid in green pepper fruits gradually increased, but those of linoleic acid and linolenic acid decreased, and that calcium chloride treatment alleviated the decrease in PC, PE, PI, PG, MGDG, DGDG, linoleic acid, and linolenic acid, and delayed the increase in PA, O-LysoPE, lysophosphatidylcholine (LPC), palmitic acid, and stearic acid. In addition, Ma et al. (2020) found that methyl jasmonate treatment raised the contents of PC, PE, and phosphatidylserine (PS) in sweet peppers, but lowered the PA and DGDG contents, implying that MeJA treatment could reduce CI by reducing membrane lipid damage.

### 3.2 Effects of CI on ROS scavenging system

The reactive oxygen species in plant cells is a byproduct of biotic and abiotic stresses. The excessively accumulated ROS including hydroxyl radicals (-OH), superoxide radicals (O_2_^-^), singlet oxygen (^1^O_2_), and hydrogen peroxide (H_2_O_2_) will attack membrane lipids, deoxyribonucleic acid (DNA), and proteins, thus leading to membrane system damage, accumulation of toxic metabolites, metabolic disorders, and even cell death (Lu et al., 2020). ROS metabolism is controlled by a well-coordinated system based on non-enzymatic (phenols, glutathione, ascorbic acid, flavonoids, and others) and enzymatic (catalase (CAT) antioxidants, peroxidase (POD), superoxide dismutase (SOD), ascorbate peroxidase (APX), and other enzymes. It is worth noting that ascorbic acid and glutathione are one of the small molecule antioxidants in cells, which can directly react with ROS to remove ROS, or they serve as enzyme substrate to assist ROS removal (Wang and Zhu, 2017), implying that the antioxidant systems in fruit and vegetable tissues need to coordinate with each other to keep ROS at a relatively low level. Enhanced ascorbate-glutathione (AsA-GSH) cycle has been shown to inhibit the increase in hydrogen peroxide content and alleviate CI in bell pepper fruit (Endo et al., 2019). Kantakhoo et al. (2022) found that during storage at 1°C, the antioxidant capacity of eggplant decreased, but its H_2_O_2_ level increased. This suggests that maintaining the activity of antioxidant enzymes and maintaining the content of AsA, etc. can scavenge excessive ROS, and help to alleviate the cold damage of fruits and vegetables during postharvest cold storage.

Excessive accumulation of reactive oxygen species also leads to lipid peroxidation of cell membranes. Malondialdehyde (MDA) is the end product of cell membrane lipid peroxidation, and its content is closely related to the severity of CI, with higher MDA content suggesting more severe CI. It has been reported that on day 20 post cold storage, sweet pepper exhibited more obvious CI symptoms and more MDA accumulation under 4°C storage than under 10°C storage (Zhang X. Y. et al., 2021). According to Shu et al. (2022b), ethylene treatment increased the antioxidant enzyme activity of tomatoes, inhibited the massive accumulation of MDA, exhibiting a desirable inhibitory effect on membrane lipid peroxidation within the cell membrane, thereby enhancing the cold resistance of tomatoes. These results suggest that decreased scavenging capacity of free radicals such as reactive ROS, which cause excessive accumulation of ROS in cells and oxidative damage to cell membranes and other biomacromolecules, may be one of the most important reasons for the occurrence of CI in solanaceous fruits and vegetables.

### 3.3 Effects of CI on material metabolism

Organic acids, soluble sugars, amino acids, pigments, and other aroma compounds constitute the unique flavor of fruits and vegetables. Sugar plays a key role, and any changes in sugar will lead to changes in flavor, sweetness, and sourness. Sugar metabolism is an important process for the post-harvest deterioration of fruit and vegetable quality. Studies have indicated that treatment with trypsin before cold storage effectively promotes sugar metabolism and carotenoid biosynthesis, maintains the postharvest quality of green pepper fruits, and reduces CI during cold storage (Zhao et al., 2024). During the ripening process of tomato fruit, the overexpression of *SlBRI1* promotes the increase in the levels of soluble solids, soluble sugars, and ascorbic acid, and enhances fruit firmness, suggesting that *SlBRI1* overexpression-mediated increase in brassinosteroids (BR) signal transduction can promote fruit ripening, improve fruit quality, and alleviate CI symptoms (Shuai et al., 2022). As well, Wang F. et al. (2019) found that exogenous melatonin reduced decay and weight loss rates and increased TSS and TA levels, suggesting that by reducing the degradation rate of nutrients (soluble sugars and TA), ultimately fruit CI can be delayed and fruit quality can be maintained.

Phenolic substances are also abundant key secondary metabolites in fruits and vegetables, which can further induce antioxidant activity during cold stress. In eggplant, it has been proved that the higher levels of total flavonoid and total phenolic contributed to alleviating CI symptoms under cold stress (Barzegar et al., 2021). Likewise, the anthocyanin levels in the pericarp dramatically decreased in the control and melatonin-treated pericarp, but were higher in the melatonin-treated pericarp from 5 d to 25 d (Song et al., 2022). The accumulation of phenolic substances is directly related to the accumulation of endogenous melatonin during cold storage.

### 3.4 Effects of CI on respiration metabolism

Fruits and vegetables are still living organisms after harvest, and respiration is one of their most basic physiological processes, and they need to respire to maintain normal life activities. However, excessive respiration consumes a large amount of organic matter, causing their quality to decline, accelerating aging and shortening their shelf life. Low temperature can induce changes in the respiratory system and metabolic pathways in fruits and vegetables. The respiratory metabolism of solanaceous fruits and vegetables is abnormal mainly because: (1) the normal enzymatic metabolism is dysregulated during low-temperature storage, which makes the respiratory efficiency reduced; (2) anaerobic respiration makes the cell produce toxic and harmful substances (acetaldehyde, ethanol, etc.). At the same time, the accelerated respiration leads to energy deficit, which weakens the ability of solanaceous fruits and vegetables to resist cold damage, resulting in cold damage symptoms. Such as, Alaboudi et al. (2024) stored red bell pepper at 4°C and found that extended storage duration led to a significant increase in cells respiration rate. Song et al. (2022) found that melatonin treatment could inhibit the respiration rate of eggplant to maintain the quality of eggplant and reduce CI damage. Moreover, the longer the time when tomatoes are stored at 4°C, the more obvious the odor produced after they are put back to 22°C environment. After continuous 12-day cold storage at 4°C, the acetaldehyde and ethanol contents in the tomato fruits were almost doubled, compared with those in the tomato fruits stored at 22°C, indicating that cold storage has a negative impact on the overall flavor of tomatoes. At the same time, it also aggravated the CI to the fruit (Farneti et al., 2015).

### 3.5 Effects of CI on endogenous hormones

The cold tolerance of plants is closely related to the contents of their endogenous hormones. The regulation of fruit cold tolerance by plant hormones is a complex process, and this regulation process depends not only on the level of endogenous hormones, but also on the synergistic/antagonistic effects of various hormones in the signal transduction pathway. As an important endogenous hormone in plants, abscisic acid (ABA) plays a vital role in responding low temperature stress. In low-temperature environments, the activation of ABA biosynthesis results in its continuous accumulation, thus leading to the formation of a complex between the PYR/PYL/RCARS receptor and protein phosphatase 2C (PP2C) in the ABA signal transduction pathway. The formed complex activates phosphorylated sucrose non-fermenting-1-related protein kinase 2 (SnRK2), further triggering transcription factor bZIP and initiating the expression of stress-related downstream genes (Liu Y. D. et al., 2023). As key endogenous hormone in plants, Gibberellins (GA) are extremely important for promoting plant growth and development. The content of endogenous GA in refrigerated fruits is lower than that stored at room temperature, and the reduction in bioactive GA content is related to the expression down-regulation of GA biosynthetic genes *GA20oxl* and *GA30oxl* (Pusittigul et al., 2012). It has been shown that 15-min treatment with 0.2 mM GA solution followed by storage at 4°C reduced the loss of electrolytes and lowered MDA content, thus increasing proline content in tomatoes and enhancing oxidase activities (SOD, CAT and POD), ultimately mitigating low-temperature damage to tomato fruits (Ding et al., 2015).

In addition to ABA and GA, auxin, ethylene, salicylic acid and BR are also endogenous hormones in fruits and vegetables. Auxins, a critical plant hormoneare,abbreviated as IAA, whose chemical nature is indoleacetic acid. IAA plays a vital role in the response to cold stress, and previous studies have suggested that IAA levels are linked to cold resistance in plants (Rahman, 2013). For example, an increase in endogenous IAA content enhanced the cold resistance (Jankauskiene et al., 2022). The involvement of endogenous IAA in inducing seed browning in cold-sensitive pepper fruits was first reported by Seo et al. (2023). Zheng et al. (2023) observed a decrease in IAA levels throughout the storage period in response to 1-MCP treatment, which suggested a link between IAA biosynthesis and 1-MCP induced chilling tolerance. Ethylene plays a regulatory role in the low temperature stress response. The endogenous ethylene biosynthesis has been reported to positively regulate the cold tolerance of tomato fruit, but the partial loss of ethylene signal transduction function will aggravate CI (Yu et al., 2019). One previous study demonstrated that tomato fruits treated with exogenous ethylene showed lower CI symptoms, CI incidence, and CI index, and the reduced MDA accumulation and ion leakage (Shu et al., 2022a, 2022b).

SA is an endogenous small molecule phenolic compound ubiquitously present in plants, and its content increased significantly under low temperature conditions, and if the concentration of SA was too high it was unfavorable for the growth of plants, while the low concentration of SA had a promotional effect on the expansion and growth of cells. SA can scavenge reactive oxygen species under low-temperature stress, which is a way to regulate the stress response by increasing the content of non-enzymatic antioxidants and the activity of antioxidant enzymes (Wang W. L. et al., 2019). It has been found that endogenous SA in pepper fruits increased gradually during storage at 2°C, and that there was a significant difference in SA content after 10 d and 25 d of storage at 2°C and 13°C, even when the symptoms of cold damage were pronounced (Seo et al., 2020). Ding et al. (2016) have shown that SA might protect tomato fruit from CI and oxidative damage through regulating GA metabolism, *CBF1* gene expression, and antioxidant enzyme activities.BR is polyhydroxylated steroidal phytohormones with many important roles in plant nutritional and reproductive growth as well as in the response to abiotic stresses (Kono and Yin, 2020). Endogenous BR content rises in plants during cold stress. And the ability of BR to enhance cold tolerance is reflected in its ability to extend the storage time of fruits by reducing the degree of CI suffered during cold storage. Studies have shown that in tomato, overexpression of the BR biosynthesis gene *SlCYP90B3* (*DWARF*) improves chilling tolerance in fruit by increasing *SlCBF1* expression and antioxidant enzyme activity (Hu et al., 2022).

### 3.6 Effects of CI on permeable substance

Under low-temperature stress, the content of osmoregulatory substances in plants can change, which can stimulate the formation of various osmoregulatory capacities in plants, thus improving the resistance of plants to low temperature. Protein can serve not only as osmosis-regulating substance to improve the stress resistance of plants, but also as nutrients for plants. As an important component of cells, protein is directly involved in cellular metabolic enzyme synthesis. The study showed that ferulic acid is a derivative of hydroxycinnamic acid, which up-regulates the expression of genes related to the C-repeat binding factor (CBF) transcriptional regulatory pathway, thus promoting the accumulation of soluble protein and proline, ultimately enhancing tomato fruit tolerance to storage at 4°C (Shu et al., 2022b). The functions of protein *SlCML37* in tomato are similar to those of calmodulin, serving as a calcium ion sensor and interacting with the proteasome maturation factor *SlUMP1*, hence enhancing the cold tolerance of tomato fruits (Tang et al., 2021).

Proline (Pro) is a highly hydrophilic compound, playing an important role in stabilizing intracellular metabolic processes. Its accumulation and degradation are a dynamic equilibrium process. Pro can regulate the lipid permeability of cell membranes under low temperature stress by modulating the activity of related enzymes, including 1-pyrroline-5-carboxylate synthetase (P5CS), ornithine aminotransferase (OAT), and proline dehydrogenase (ProDH). Wang F. et al. (2022) reported that treatment of green bell pepper with OA led to increasing the accumulation of proline by enhancing the activity of P5CS and OAT, and decreasing the activity of ProDH eventually improving the cold tolerance of green peppers and alleviating CI. Furthermore, BR treatment increases the accumulation of proline in tomato fruit, hence inhibiting CI symptoms and enhancing its cold resistance (Bai et al., 2021).

Polyamines are one of the most important compounds related to plant stress resistance, and they can reduce CI, stabilize cell membranes, and prevent oxidation. The regulation on polyamine synthesis and catabolism is the key to improving plant stress resistance. The diamine oxidase (DAO) and polyamine oxidase (PAO) are responsible for the degradation of polyamines, while arginine decarboxylase (ADC) and ornithine deacetylase (ODC) are responsible for formation of putrescine (Yi et al., 2024). The related studies have demonstrated that exogenous melatonin can upregulate the proline-producing P5CS and OAT genes, thus enhancing cold tolerance of tomatoes and increasing the contents of ODC and ADC (Aghdam et al., 2019). Thus, polyamines can act as excitons to stimulate stress-protective responses and compensate for the negative effects of low-temperature stress.

## 4 Molecular mechanisms

**Figure 3:**
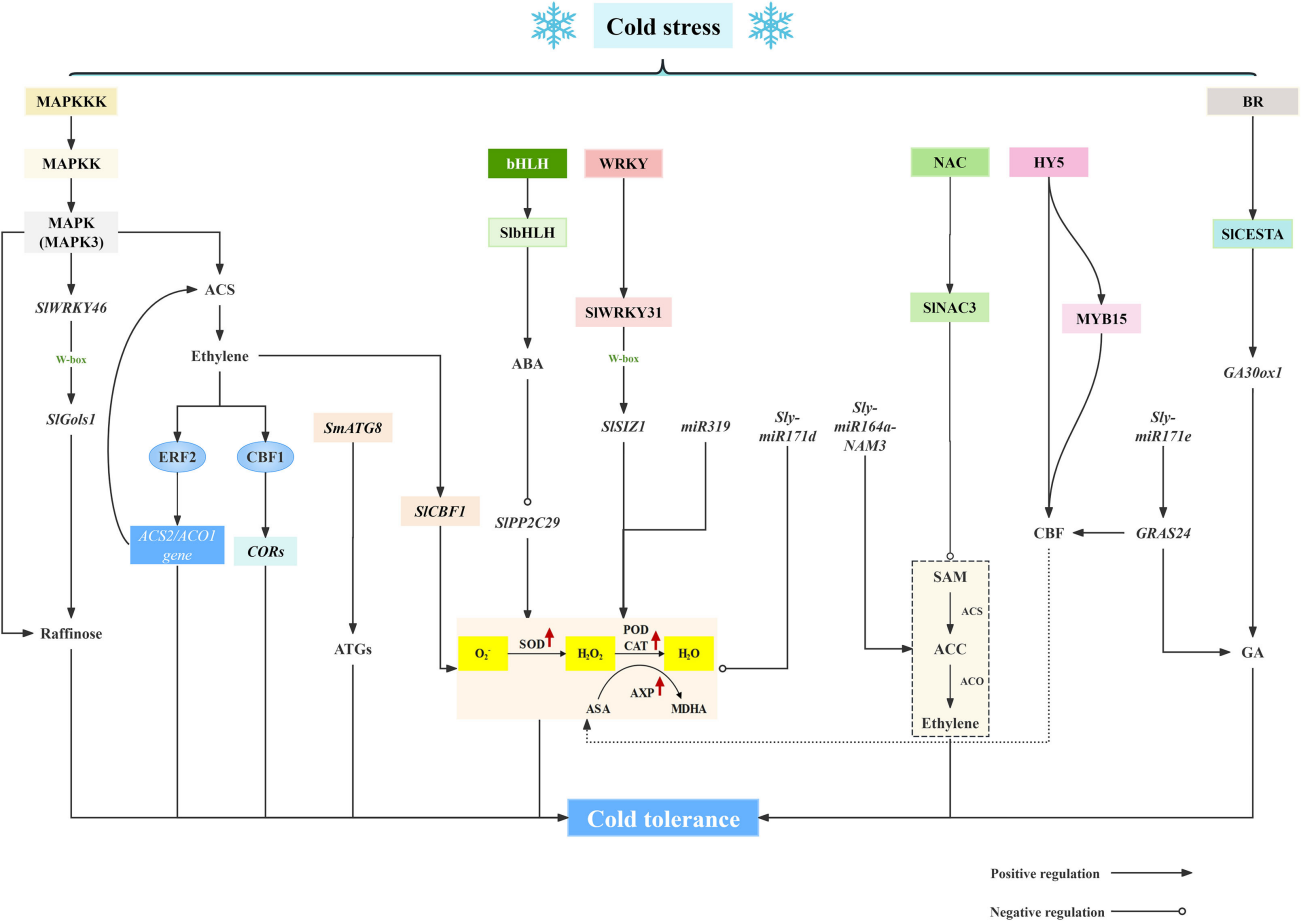
Molecular mechanisms of cold injury in solanaceous vegetables and fruits.

Analyzing cold stress-related gene expressions and deciphering CI resistance mechanisms in solanaceae will lay the foundation for inducing cold tolerance and preventing and controlling CI during low-temperature storage. Low temperature modulates the expression and regulation of genes related to cold stress, affecting both transcription and translation processes, as shown in **Figure 3**. In recent studies, the explorations of molecular mechanisms underlying fruit and vegetable response to cold stress are mainly focused on the potential mechanisms of CI reduction and the identification of the specific molecules mediating plant adaptation to low temperatures at the transcriptional and protein levels, which provides new insights into the generation of plant cold resistance.

### 4.1 CI-related signal transduction

Exposing plants to low temperatures can trigger a highly complex regulatory phenomenon that causes up-regulation or down-regulation of genes which altogether ends up providing a chilling tolerance response in the plant. The cold response process includes kinases and proteases, plant hormones, and transcription factors, which together constitute the cold-responsive regulatory pathway (Pandey and Gayen, 2024). Mitogen-activated protein kinase cascade (MAPK) is a vital pathway in intracellular signal transduction. MAPK signaling cascades are essential for various physiological responses, for instance, plant growth and development, stress response, etc (Furuya et al., 2014). The MAPK signaling cascade involves three types of proteins: MAPK kinase kinase (MAPKKK), MAPK kinase (MAPKK), and MAPK (Xu and Zhang, 2015). It has been confirmed that MAPK plays a significant role in cold response in plants. At low temperatures, the expression of MPK3 was rapidly induced in *Arabidopsis*, leading to the activation of the kinase activities of MPK4 and MPK6 (Zhao et al., 2017). Such as, Shu et al. (2024) results reveal that overexpression of SlMAPK3 promoted the accumulation of galactinol and raffinose under cold stress. Moreover, SlMAPK3 promoted the expression of *SlWRKY46* at low temperatures and interacted with *SlWRKY46* protein. Overexpression of *SlWRKY46* enhanced cold resistance. Furthermore, *SlWRKY46* directly bound to the promoter of *SlGols1* to enhance its expression and promoted the accumulation of raffinose. Also, Anti et al. reported that SlMAPK3 positively regulates ethylene production in fruit and is involved in ethylene-mediated cold tolerance in postharvest tomato (Shu et al., 2023). Zhao et al. (2013) indicated that MAPK cascade as an upstream regulator of ACS activity and ethylene production regulates the expression of downstream genes. The up-regulated ACS and ACO gene expressions increase ethylene production under cold stress. Transcription factors such as CBF1 interact with DRE to induce the expression of cold responsive (COR) genes. The changes regulated by MAPK cascade improve cold tolerance of tomato fruit. Additionally, in the complex mechanism of fruit cold stress, phytohormones emerge as a class of functional molecules that can effectively regulate fruit cold tolerance (Wani et al., 2016). Mitigation of CI damage by these phytohormones could be attributed to: (1) stabilization of cell membrane structure (Singh and Roychoudhury, 2023), (2) modulation of arginine catabolism (Ding et al., 2015), (3) maintenance of ROS homeostasis (Lei et al., 2023), and (4) induction of CBF signaling pathway (Shu et al., 2022a). Phytohormones significantly affect the expression of cold-responsive genes which in turn control multiple signaling pathways and ultimately regulate fruit cold tolerance (Souza et al., 2022). Such as, ABA treatment inhibits the expression of *SIPP2C29* gene, and SlbHLH1 reduces the transcription of *SIPP2C29* by binding to the E-box recognition site in its promoter, indicating that SlbHLH1 mediates ABA to down-regulate *SIPP2C29* expression and PP2C activity, thereby reducing CI (Jiao and Sun, 2024). In addition, previous study indicated that endogenous ethylene production enhanced cold tolerance of tomato fruit by inducing C-repeat/DRE binding factor (CBF) to modulate the expression of downstream genes (Zhang and Huang, 2010). Yu et al. (2023) demonstrated that ethylene perception was beneficial for quality maintenance of postharvest tomato during cold storage, which regulated ROS metabolism, affected the accumulation of other phytohormones, and induced *SlCBF1* gene expression. These illustrates the important role of ethylene perception in mitigating CI damage and maintaining fruit quality in stored tomatoes.

### 4.2 CI-related transcription factors and key genes

There are several families of low-temperature-related transcription factors in solanaceae fruits and vegetables, such as WRKY, MYB, CBF, NAC, and these members are involved in cold tolerance in response to cold damage by regulating the expression of related defense genes. Transcription factors can markedly impact the cold tolerance of fruits by modulating the expression of key genes involved in membrane lipid metabolism. During cold storage, SlWRKY31 transactivated the *SlSIZ1* expression by recognizing the W-box binding site in its promoter. This positive transcription regulation ameliorated the oxidative damage, thus alleviating the CI in tomato fruit (Jiao et al., 2024). Wang T. et al. (2024) elaborated that SlNAC3 enhancing cold tolerance in tomatoes by suppresses ethylene biosynthesis. Meanwhile, SlCESTA has been identified as a bHLH in tomato, which is similar to the cellulose synthase (CesA) gene in *Arabidopsis thaliana*, and it has been found to be localized in the nucleus, responding to BR signaling, and overexpression of transcription factor SlCESTA affects the dynamic balance of GA and enhances the low temperature tolerance of tomato fruits (Shuai et al., 2022). Furthermore, Zhang et al. (2020) showed that HY5 can directly regulate CBF transcription and indirectly affect CBF expression through MYB15, and the synergistic effect of HY5 and MYB15 can precisely regulate CBF expression and enhance the cold tolerance of tomato. Albornoz et al. (2023) found that a transgenic phenotype overexpressing *AtCBF1* in tomato fruit was characterized, and revealed this gene influenced ripening as well as fruit’s response to postharvest cold stress. In eggplant fruit, under 4°C low-temperature stress, the expression of the *SmATG8* gene was continuously upregulated, suggesting a critical role of ATGs in protecting eggplant fruit from CI (Wang et al., 2024) In conclusion, these transcription factors in plants are actively involved in the response to low-temperature stress, and there is increasing evidence that they are important transcription factors for improving cold tolerance in plants.

### 4.3 CI-related microRNA

In plants, miRNA (microRNA) is an endogenous non-coding small molecule RNA with a length of 19~24 nucleotides, which plays a key role in the post-transcriptional gene regulatory network. The miRNA-mediated gene response is one of the potential mechanisms for plants to respond to abiotic stress (Voinnet, 2009). In recent years, the regulatory role of miRNA in plant stress response has been reported. Low temperature affects the expression of miRNA, and key miRNA genes with different expression levels are involved in the regulation of different pathways. miR319 participates in the regulation of tomato tolerance to chilling stress through reactive ROS metabolism (Shi et al., 2019). Under chilling stress, *Sly-miR164a-NAM3* in tomatoes regulates ethylene content, thus affecting the wilting and cold tolerance of tomato plants (Dong et al., 2022). For tomato fruit, silencing the expression of *Sly-miR171d* can effectively reduce ROS and affect the expression of cold regulation-related genes (Xing et al., 2022), while silencing *Sly-miR171e* upregulates the expression of the target gene *GRAS24* and increases GA content and CBF expression, hence improving the cold resistance of tomato fruit (Zhao et al., 2022).

## 5 CI control measures

**Figure 4:**
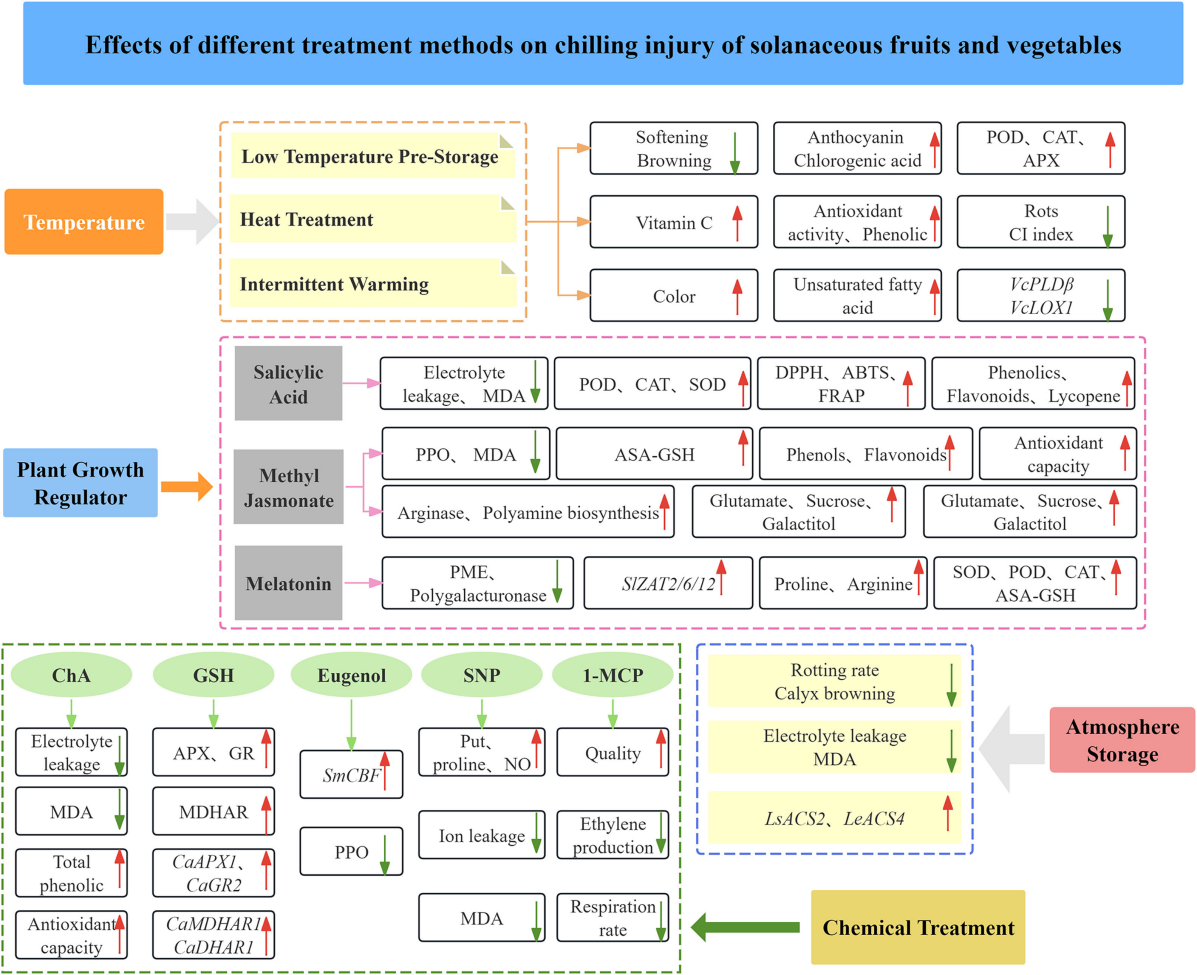
Effects of different methods on CI of solanaceous vegetables and fruits.

CI is the most common phenomenon for tropical and subtropical fruits and vegetables sensitive to low temperatures. As the CI-caused loss of fruits and vegetables increases, revealing CI formation mechanism and exploring CI control measures become increasingly important for the storage of fruits and vegetables. The multiple methods for CI control have been developed, including temperature acclimation, controlled atmosphere storage, plant growth regulators, and chemical treatments so as to reduce the incidence of CI, extend the storage period, and maintain good quality of fruits and vegetables (**Figure 4**).

### 5.1 Temperature acclimation method

#### 5.1.1 Low temperature pre-storage

Low temperature conditioning (LTC) refers to a short-term storage of cold-sensitive fruits and vegetables at the temperature above the damage threshold to stimulate their tolerance to subsequent lower temperature storage (Wang et al., 2017). LTC is an early method used for CI control of fruits and vegetables, and it has been demonstrated to effectively alleviate CI to tomatoes, eggplants, peppers, cucumbers, mangoes, papayas, and others (Chaudhary et al., 2014). The 2-day storage of eggplants at 10°C not only reduced the softening and browning, but also greatly retained anthocyanin and chlorogenic acid contents in eggplants stored at 4°C (Darré et al., 2022). Similarly, when eggplant fruits were stored at 13°C for 2 days, LTC improved the appearance of eggplants, delayed the occurrence of CI, inhibited the browning of sepals, and reduced the loss of bioactive substances (Shi et al., 2018). Wang et al. (2019) found that LTC in combination with MeJA treatment effectively maintained the quality of sweet peppers, enhanced the activity of POD, CAT and APX, and improved the tolerance of post-harvest sweet pepper fruits to CI. Overall, LTC inhibits oxidative damage to fruits and vegetables during cold storage by increasing the activity of phenolic substances and antioxidant enzymes in the fruit.

#### 5.1.2 Heat treatment before storage

Heat treatment (HT) at 38~60°C is a safe and environmentally friendly method, which has a good inhibitory effect on CI of post-harvest horticultural products. HT mainly refers to the treatment on post-harvest horticultural products with hot air (HA) or hot water (HW) for a period of time before refrigeration to delay the ripening of fruits, inhibit decay, thus delaying CI symptoms. Lopez-Velazquez et al. (2020) found that 1-minute heat treatment on sweet peppers at 53°C reduced the CI symptoms of sweet peppers, which was conducive to the stabilization of plasma membrane and the maintenance of vitamin C content. Gabriela et al. (2021) reported that immersion of green peppers in HW at 53°C (1~3 min) slowed down fruit senescence, reduced rots and CI index, maintained fruit quality, and enhanced enzymatic antioxidants. The CI index and weight loss rate of eggplants treated with HW at 45°C for 10 min were the lowest, and the antioxidant activity and total phenolic content of HW-treated eggplants were twice as much as those in the control group (Kantakhoo et al., 2022). It was reported that cell wall degradation genes were down-regulated in tomato fruits which were first subjected to HW treatment and then 2- week cold storage, and CI symptoms including water loss and wilting were mitigated after another 2-week treatment at room temperature, indicating that HW treatment is helpful for maintaining cell wall integrity and expansion during cold storage (Cruz-Mendívil et al., 2015). Salazar-Salas et al. (2017) found that hot water induction (42°C, 5 min) on tomato fruits seemed to be associated with the prevention of protein denaturation, activation of antioxidant and defense systems, and potential regulation of cold-sensitive genes.

#### 5.1.3 Intermittent warming

Intermittent warming (IW) is a potential environmentally friendly postharvest technology for reducing senescence and CI of horticultural products. IW refers to the periodic exposure of fruits to temperatures between 20°C and 27°C during storage. IW has been reported to enhance the color of tomatoes stored at 2.5°C and 6°C, reduce their senescence during cold storage, lower the rots rate, mitigate CI of tomato fruits (Biswas et al., 2012). IW can effectively decrease the CI index of sweet peppers, maintain their firmness. After two IW cycles followed by 6-day and 13-day storage, the integrity of sweet peppers was maintained without shrinkage and decay, and even the decrease in unsaturated fatty acid content was delayed by appropriate IW cycles (Liu et al., 2015). Additionally, IW treatment can also inhibit membrane lipid metabolism induced by low temperature, reduce membrane damage by inhibiting the expression of *VcPLDβ* and *VcLOX1*, thus effectively alleviating fruit softening and decay and maintaining fruit quality (Dai et al., 2020).

### 5.2 Controlled atmosphere storage

Controlled atmosphere storage is a storage method that regulates the gas composition and concentration in the storage environment of fruit and vegetable products. Controlled atmosphere storage has been reported to extend the storage life of horticultural crops, and it includes spontaneously modified atmosphere storage (MA) and artificially controlled atmosphere storage (CA). Most studies have shown that increasing CO_2_ concentration and decreasing O_2_ concentration during low-temperature storage can reduce cold damage in postharvest fruits and vegetables. Park et al. (2018) found that elevated CO_2_ concentration and reduced O_2_ concentration could reduce fruit rotting rate, increase the expression of *LsACS2*、*LeACS4* in tomato, reduce the effect of cold damage on tomato, and extend the storage period of tomato. Afolabi et al. (2023b) reported that the 24-h treatment on sweet peppers with 10% CO_2_ in combination of MA effectively maintained the quality of sweet peppers stored at 10°C and reduced CI. Interestingly, Olveira-Bouzas et al. (2021) evaluated the use of a MAP system with trays using a packaging atmosphere composition of 10% O_2_ ~ 10% CO_2_ and silica gel as an adsorbent delayed color evolution and reduced the firmness loss, and decay rate of tomato, extend the shelf-life of tomatoes stored at refrigerated temperatures and reduced CI. Therefore, the storage and transportation of solanum fruits and vegetables can be combined with gas-conditioned storage to reduce the impact of cold damage of low-temperature storage and improve the shelf life.

### 5.3 Plant growth regulator

#### 5.3.1 Salicylic acid

Salicylic acid (SA) is an endogenous phenolic growth regulator, and it participates in regulating various physiological processes of plants as well as biotic and abiotic stress responses to reduce CI, improve freezing and salt tolerances, and others. The 4 mM SA treatment on tomato fruits followed by the 40-day storage at 10°C can significantly reduce CI symptoms and increase POD activity. SA pretreatment can improve the cold tolerance of tomato fruits by regulating their gene expression and metabolic pathways (Baninaiem et al., 2016). Treatment with 1 mM SA on tomato fruits can reduce CI and oxidative damage by regulating GA metabolism and *CBF1* gene expression and enhancing the activity of antioxidant enzymes (SOD, POD and CAT) (Ding et al., 2016). In addition, studies have shown that SA in combination with TSP is more effective in alleviating CI of sweet peppers than SA treatment alone, which effectively inhibits CI-induced membrane damage, reduces electrolyte leakage and MDA content, and improves the water retention of sweet peppers (Ge et al., 2020). Baek et al. (2023) have found that the fruit quality, total phenolics, flavonoids, lycopene, β-carotene and antioxidant activity (DPPH, ABTS and FRAP) of “Kumato” tomatoes are improved after preharvest treatment with 0.5 mM SA and 0.25 mM MeJA followed by the 24-day storage at 12.5°C. In summary, the above findings indicate that SA can effectively reduce CI and oxidative damage, thus maintaining fruit quality.

#### 5.3.2 Methyl jasmonate

Methyl jasmonate (MeJA) is a derivative of jasmonic acid (JA) and a very important lipid growth regulator in plants. MeJA is involved in regulating the growth and development of plants and the responses to environmental factors, such as anti-CI response and anthocyanin accumulation. Postharvest application of MeJA improves fruit quality mainly by promoting the production of phenols, flavonoids, raising antioxidant capacity, and delaying senescence (Wang Y. et al., 2022; Wang Y. S. et al., 2021). One study has indicated that MeJA reduces CI by delaying the maturation of antioxidant compounds, and it also prevents CI by inhibiting polyphenol oxidase activity, reducing MDA contents, and maintaining cell membrane fluidity (Chen et al., 2021). Seo et al. (2020) treated peppers with 50 μM MeJA, which inhibited the seed browning of pepper fruits during cold storage and increased the content of glutamate, sucrose, and galactitol in peppers, thereby reducing CI. Another study demonstrated that MeJA treatment followed by the 6-day storage at 4°C effectively inhibited the signaling pathway of the transcription factor *MYC2-JA*, enhanced the AsA-GSH cycle, reduced membrane lipid loss, suppressed cell wall disintegration, and activated the CMAT-CBF-ICE pathway, thus reducing the CI of sweet pepper (Fu et al., 2022). Liu J. et al., (2023) reported that 0.05 mM MeJA treatment inhibited CI by regulating the reactive oxygen metabolism, polyamine and JA signaling pathways, and low temperature response pathways of cold-stored tomatoes. S*l*MYC2 regulates polyamine biosynthesis, participates in MeJA-induced expressions of *SlARG1, SlARG2, SlADC*, and *SlODC*, modulates the activities of arginase, ADC, and ODC, thereby improving the cold tolerance of tomatoes (Min et al., 2021). In addition, the cold tolerance of tomatoes can also be improved by regulating sugar metabolism (Zhou et al., 2021). These studies collectively reveal that 0.05 mM MeJA can induce the increase in arginase and polyamine biosynthesis, and antioxidant enzyme activity (SOD, CAT and POD) so as to reduce CI symptoms during solanaceae cold storage.

#### 5.3.3 Melatonin

Melatonin (MT) is involved in physiological processes and regulates gene expression in plant hormone biosynthesis/decomposition pathways. MT treatment can prevent membrane lipid peroxidation and stabilize high percentages of unsaturated fatty acids and saturated fatty acids, thus maintaining cell structure and functions, eventually delaying the occurrence of CI during cold storage of fruit and vegetable horticultural products (Charoenphun et al., 2024). MT treatment can also enhance the antioxidant defense system of plants by increasing the activity of SOD, POD, CAT, enhancing AsA-GSH cycle, and improving the scavenging ability of ROS, thereby protecting plants from oxidative damage caused by low temperature stress (Wang Y. et al., 2022). Kong et al. (2020) demonstrated that MT treatment increased the transcription level of antioxidant enzyme-related genes in sweet peppers and raised the content of proline, hence effectively reducing the CI to sweet peppers. Charoenphun et al. (2024) investigated different concentrations of MT treatment, and found that a high concentration (0.1 mM) of MT could improve the quality of pepper fruits and their resistance to low temperature stress, thus reducing physicochemical losses. At the same concentration (0.1 mM), MT could reduce CI and maintain the quality of post-harvest fruit by improving the antioxidant capacity of cold-stored eggplant and inhibiting the activity of cell wall-degrading enzymes such as pectin methylesterase (PME) and polygalacturonase, thereby extending fruit shelf life (Song et al., 2022). In addition, MT can also activate *CBF1* gene expression and arginine pathway activity by upregulating *SlZAT2/6/12* to endow tomato fruit with cold tolerance (Aghdam et al., 2019).

### 5.4 Chemical treatment

Various chemical treatment techniques have been widely explored and applied to improve the cold tolerance of fruits and vegetables, which are essential for ensuring the quality of fruits during storage. Studies have shown that treatment with 1-methylcyclopropene (1-MCP) can extend the life of tomato fruits and maintain their quality during storage, the ethylene production and respiration rate were significantly reduced (Bahar et al., 2022). Zhao et al. (2011) found that treatment with 0.02 mM sodium nitroprusside (SNP) on tomatoes reduced their CI index, MDA content, and ion leakage level, that SNP treatment protected tomatoes from CI damage by inducing nitric oxide (NO) accumulation and *LeCBF1* expression, and that the 0.5-minute pretreatment with 0.2 mM exogenous arginine at -35 kPa followed by the 28-day storage at 2°C resulted in CI alleviation of cold-stored tomato fruits by enhancing the concentration of endogenous putrescine, proline, and NO, which might be mainly due to the increase in arginase, ADC, ODC, OAT, and nitric oxide synthase (NOS) enzyme activities (Zhang et al., 2013). In addition, treatment with 25 μL/L eugenol on eggplants followed by the 21-day storage at 4°C weakened the activity of polyphenol oxidase, maintained the contents of soluble solid and proline, and induced the upregulation of *SmCBF* genes, thereby enhancing the cold tolerance of eggplant fruits (Huang et al., 2019). Zhang X. Y. et al. (2021) demonstrated that Ca^2+^ treatment effectively reduced the CI of green peppers and maintained their quality. And Yao et al. (2021) results showed that exogenous GSH treatment could alleviate CI in pepper fruits during cold storage by triggering the AsA-GSH cycle and improving the APX, GR, and MDHAR levels, upregulated *CaAPX1, CaGR2, CaMDHAR1*, and *CaDHAR1*. Chlorogenic acid (ChA) treatment can also alleviate the softening of tomato fruits, reduce the accumulation of MDA and electrolyte leakage, maintain membrane integrity, thus reducing CI damage in tomatoes. In addition, higher total phenolic content (TPC) was maintained, indicating better antioxidant capacity and better preservation of fruit quality in ChA treatment (Ilea et al., 2024).

### 5.5 Biotechnology

In recent years, with the continuous progress of molecular biology technology, the transcriptional regulation mechanism of plants under low-temperature adversity has received extensive attention. Cold-resistant genes are induced by molecular mechanisms under low-temperature stress to produce cold-resistant plants to adapt to the low-temperature environment. Song et al. (2021) identified *ShPP2-1* as a novel gene expressed by cold-induced expression that negatively regulates cold tolerance by intensifying cell membrane damage when plants experience cold stress, and showed that the combination of low expression levels of *PP2-1* and high expression levels of *ACR11A* can enhance cold tolerance of tomato. Song et al. (2023) study shows that SlBBX17 can enhance SlHY5 protein stability and form a complex with SlHY5 to activate the transcription of *SlCBF* genes, eventually positively regulating tomato cold tolerance. In the same way, being a key regulator of JA signaling, MYC2 activates the transcription of *SlERF.B8*, which potentiates the output of JA signaling, eventually reinforcing cold tolerance in tomato (Ding et al., 2022). Furthermore, in eggplants, the overexpression of the *R2R3 MYB* transcription factor gene *SmMYB1* led to transgenic eggplants that accumulate higher concentrations of anthocyanin in the fruit peel and flesh (Zhang and Jie, 2016). Thus, the expression of all these genes is closely related to cold tolerance in tomato.

## 6 Future research prospects

Temperature is an important factor affecting the post-harvest storage quality of solanaceous fruits and vegetables. Low-temperature storage is one of the most effective methods for preserving solanaceae, but low temperature in the process of storage and transportation can easily cause irreversible CI to the fruit cell tissues, thus resulting in quality decline, shortened shell life period, and huge economic losses. CI is also an obstacle to the healthy development of the solanaceae industry. Therefore, alleviating CI symptoms in solanaceous fruits and vegetables and exploring the optimal storage measures to control CI are of great significance for maintaining the quality of solanaceous fruits and vegetables and improving their economic benefits. This paper reviews the physiological and biochemical mechanisms, molecular mechanisms, and regulatory measures of CI in solanaceae caused by low-temperature storage. Further studies are suggested to be conducted from the following perspectives in the future: (1) The synergistic mechanisms of combined cold resistance treatments should be investigated (1-MCP in combination with HT, 1-MCP combined with intermittent warming, etc) (2) New biological and food technologies such as low-temperature plasma, pulse electric field, and nano-packaging should be applied to strengthen the flavor quality control of solanaceae during low-temperature storage. The standardized management can be implemented to improve the safety and commercial feasibility of solanaceous products; (3) It is worthwhile to explore the pathways of key genes and proteins in response to low temperature stress in solanaceous fruits and vegetables so as to improve the network of solanaceae cold resistance regulation. Finally, solanaceae varieties with stronger cold resistance should be bred from a genetic perspective.
